# Characterization of the complete chloroplast genome of *Allium mongolicum*

**DOI:** 10.1080/23802359.2020.1756951

**Published:** 2020-05-12

**Authors:** Hongxia Wang, Fahui Ye, Xiang Li, Lirong Wang, Jiuli Wang, Wenjie Chen

**Affiliations:** a Key Laboratory of Biotechnology and Analysis and Test in Qinghai-Tibet Plateau, College of Ecological Environment and Resources, Qinghai Nationalities University, Xining, People’s Republic of China; b Qinghai Provincial Key Laboratory of Crop Molecular Breeding, Key Laboratory of Adaptation and Evolution of Plateau Biota, Northwest Institute of Plateau Biology, Innovation Academy for Seed Design, Chinese Academy of Sciences, Xining, Qinghai, People’s Republic of China

**Keywords:** *Allium mongolicum*, chloroplast genome, phylogenetic tree

## Abstract

*Allium mongolicum* is a kind of wild vegetable with high nutritional value and even a traditional Chinese medicine. Here, we reported the complete chloroplast genome sequence of *Allium mongolicum*. The size of the chloroplast genome is 153,376 bp in length, including a large single copy region (LSC) of 82,912 bp, a small single copy region (SSC) of 18,054 bp, and a pair of inverted repeated regions of 26,205 bp. The *Allium mongolicum* chloroplast genome encodes 115 genes, including 69 protein-coding genes, 38 tRNA genes, and eight rRNA genes. Phylogenetic tree showed that *Allium mongolicum* is closely related to *Allium przewalskianum.*

*Allium mongolicum* Regel, also known as the Mongolia leek, is a perennial and xerophytic herb. It grows in high altitude desert steppe and desert areas (Wang et al. 2013), mainly found in desert land in Qinghai, Gansu, Xinjiang and Inner Mongolia (Muqier et al. 2017). *A. mongolicum* has a higher nutritional value and medicinal value. Its ecological functions of sand-fixing, with good development prospects, cannot be underestimated (Wang et al. 2014). In this study, we assembled the complete chloroplast (cp) genome of *A. mongolicum* (Genbank accession number: MN519208) to provide genomic and genetic sources for further research.

The fresh leaves of *A. mongolicum* were collected from Gonghe (100.26E, 36.26 N), Qinghai Province, China. Total genomic DNA of *A. mongolicum* was extracted from leaf tissues with the modified CTAB method (Doyle and Doyle 1987). The voucher specimen was deposited in Herbarium of the Northwest Institute of Plateau Biology (HNWP, whx2019003), Northwest Institute of Plateau Biology, Chinese Academy of Sciences. Genome sequencing was achieved on the Illumina HiSeq Platform (Illumina, San Diego, CA) at Gene pioneer Biotechnologies Inc., Nanjing, China, and 6.92 GB of sequence data was generated. The trimmed reads were assembled via NOVOPlasty (Dierckxsens et al. 2017). The assembled genome was annotated using Plann v1.1 (Huang and Cronk 2015) and the annotation was corrected with Geneiousv11.0.3 (Kearse et al. 2012).

The chloroplast genome of *A. mongolicum* was 153,376 bp in length, containing a large single copy region (LSC) of 82,912 bp, a small single copy region (SSC) of 18,054 bp, and a pair of inverted repeat (IR) regions of 26,205 bp. Genome annotation predicted 115 genes, including 69 protein-coding genes, 38 tRNA genes, and eight rRNA genes. The overall GC-content of the chloroplast genome was 36.06%, with the corresponding values in the LSC, SSC, and IR regions were 34.63%, 29.40% and 42.72%, respectively.

Phylogenetic analysis suggested that *A. mongolicum* is closely clustered with *A. przewalskianum* (Figure 1), which was generated based on the 25 complete cp genomes. Alignment was conducted using MAFFT (Katoh and Standley 2013). The phylogenetic tree was built usingMEGA7 (Kumar 2016) with bootstrap set to 10,000. This study could lay a foundation for chloroplast genome engineering of *A. mongolicum* in the future.

**Figure 1.**
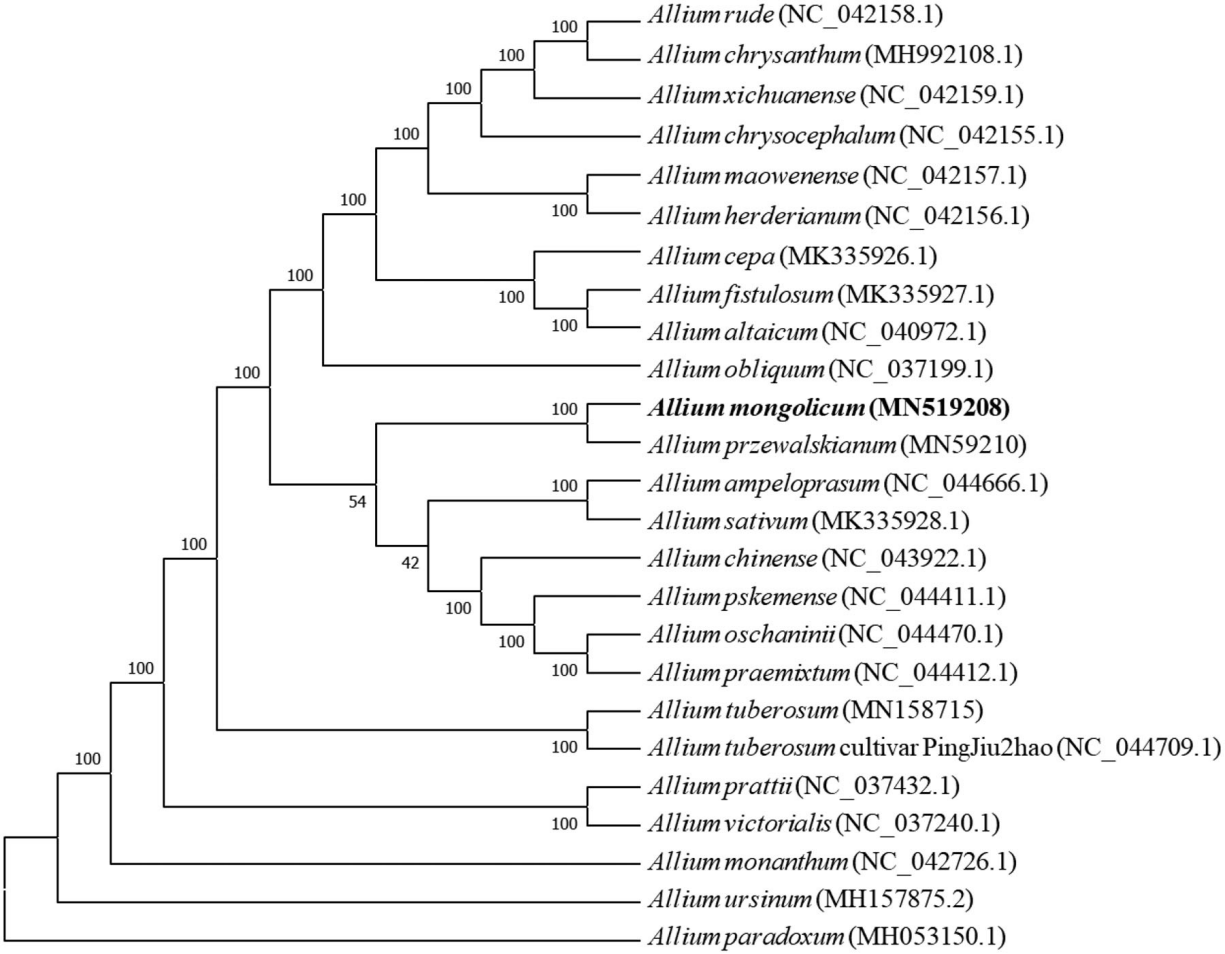
The NJ tree based on 25 chloroplast genomes.

## Data Availability

The data that support the findings of this study are openly available in NCBI at https://www.ncbi.nlm.nih.gov/, reference number [MN519208], or available from the corresponding author.
